# Assessing the coverage of full antenatal care among adolescent mothers from scheduled tribe and scheduled caste communities in India

**DOI:** 10.1186/s12889-023-15656-1

**Published:** 2023-05-01

**Authors:** Aditya Singh, Vineet Kumar, Harpreet Singh, Sourav Chowdhury, Sanjana Sharma

**Affiliations:** 1 Department of Geography, Institute of Science, Banaras Hindu University, Varanasi, Uttar Pradesh India; 2 External Research Collaborator, Girl Innovation, Research and Learning (GIRL) Centre, Population Council, New York, USA; 3 Department of Geography, Raiganj University, Raiganj, West Bengal India

**Keywords:** Antenatal care, Iron and folic acid, Scheduled Caste (SC), Scheduled Tribe (ST), Maternal health, National Family Health Survey-4, India

## Abstract

**Background:**

The persistently high rates of maternal mortality and morbidity among historically marginalised social groups, such as adolescent Scheduled Castes (SCs) and Scheduled Tribes (STs) in India, can be attributed, in part, to the low utilisation of full antenatal healthcare services. Despite efforts by the Indian government, full antenatal care (ANC) usage remains low among this population. To address this issue, it is crucial to determine the factors that influence the utilisation of ANC services among adolescent SC/ST mothers. However, to date, no national-level comprehensive study in India has specifically examined this issue for this population. Our study aims to address this research gap and contribute to the understanding of how to improve the utilisation of ANC services among adolescent SC/ST mothers in India.

**Data and methods:**

Data from the fourth round of the National Family Health Survey 2015–16 (NFHS-4) was used. The outcome variable was full antenatal care (ANC). A pregnant mother was considered to have ‘full ANC’ only when she had at least four ANC visits, at least two tetanus toxoid (TT) injections, and consumed 100 or more iron-folic acid (IFA) tablets/syrup during her pregnancy. Bivariate analysis was used to examine the disparity in the coverage of full ANC. In addition, binary logistic regression was used to understand the net effect of predictor variables on the coverage of full ANC.

**Results:**

The utilisation of full antenatal care (ANC) among adolescent SC/ST mothers was inadequate, with only 18% receiving full ANC. Although 83% of Indian adolescent SC/ST mothers received two or more TT injections, the utilisation of the other two vital components of full ANC was low, with only 46% making four or more ANC visits and 28% consuming the recommended number of IFA tablets or equivalent amount of IFA syrup. There were statistically significant differences in the utilisation of full ANC based on the background characteristics of the participants. The statistical analysis showed that there was a significant association between the receipt of full ANC and factors such as religion (OR = 0.143, CI = 0.044–0.459), household wealth (OR = 5.505, CI = 1.804–16.800), interaction with frontline health workers (OR = 1.821, CI = 1.241–2.670), and region of residence in the Southern region (OR = 3.575, CI = 1.917–6.664).

**Conclusion:**

In conclusion, the study highlights the low utilisation of full antenatal care services among Indian adolescent SC/ST mothers, with only a minority receiving the recommended number of ANC visits and consuming the required amount of IFA tablets/syrup. Addressing social determinants of health and recognising the role of frontline workers can be crucial in improving full ANC coverage among this vulnerable population. Furthermore, targeted interventions tailored to the unique needs of different subgroups of adolescent SC/ST mothers are necessary to achieve optimal maternal and child health outcomes.

**Supplementary Information:**

The online version contains supplementary material available at 10.1186/s12889-023-15656-1.

## Introduction

The Indian population can  be divided into two broad groups: the mainstream and indigenous populations. The caste system, a rigid and discriminatory social stratification system, primarily governs the mainstream population. This caste system divides society into distinct hierarchical castes, including *Brahmins* (priests and scholars), *Kshatriyas* (warriors and rulers), Vaishyas (merchants and traders), and *Shudras* (peasants and labourers) [1, 2]. The caste system, which is determined by birth and restricts social mobility, has perpetuated discrimination and exclusion of lower castes, particularly the *Shudras* and *Dalits*, for centuries [3–5]. This, in turn, has resulted in socioeconomic disadvantages, limited access to education and employment opportunities, and limited political representation for these communities [3–8].

The mainstream Indian population is currently divided into Scheduled Castes (SCs), Other Backward Classes (OBCs), and Others. Those at the bottom of the social hierarchy, who have been subjected to centuries of discrimination, oppression, and abuse from those higher up in the hierarchy, are known as Scheduled Castes [6, 9, 10]. Other Backward Classes (OBCs) are socioeconomically and educationally disadvantaged communities, but their position in the social hierarchy is slightly higher than SCs. The remaining caste groups, which form the hierarchy’s top order, are grouped into a residual category called ‘Others’ [6, 8–11].

The indigenous population of India, primarily composed of tribal communities, is officially referred to as Scheduled Tribes (STs). Their categorisation is based on their unique cultural practices and geographical isolation rather than their placement in the caste hierarchy [12]. STs continue to be one of India’s most marginalised and disadvantaged communities, having faced several social, economic, and political challenges throughout history [2, 10, 11, 13]. SCs and STs comprise approximately 25% of India’s population, according to the 2011 Census of India [14]. Thus, the entire population of India is officially divided into four social groups: SC, ST, OBC, and Other.

The lasting impacts of past discrimination, restricted access to resources and opportunities, and current socioeconomic and economic disparities are reflected in the socioeconomic, demographic, and health outcomes of SC/ST communities [15]. SC/ST population frequently experience higher rates of preventable diseases, lower life expectancy, and higher maternal and infant mortality rates than other population groups. [16]. For instance, life expectancy at birth demonstrates a significant disparity across social groups, with SCs having the lowest life expectancy of 63.1 years and Others having the highest life expectancy of 68 years [17]. Additionally, SC/ST women are more likely to experience maternal morbidity and mortality than women from other social groups [16]. These maternal deaths are largely preventable if adequate and timely antenatal, delivery, and postpartum care is provided to mothers.

Antenatal care (ANC) is an essential aspect of maternal healthcare as it enables providers to identify and manage potential risks to the mother and foetus. It also offers an opportunity for women to receive information on healthy pregnancy practices and facilitates the early detection and treatment of pregnancy-related health issues [2, 18–20]. However, there is a debate about the optimal number of ANC visits for a pregnant woman. The Global Strategy for Women’s, Children’s, and Adolescents’ Health (2016–2030) Monitoring Framework recommends a minimum of four ANC visits [21], while the World Health Organization (WHO) suggests at least eight visits to lower perinatal mortality and enhance the quality of care for women [22]. Generally, a woman is considered to have received “full” ANC if she has had four or more ANC visits, at least two tetanus toxoid (TT) injections, and 100 iron-folic acids (IFA) tablets or an equivalent amount of IFA syrup during her pregnancy. While this definition is not exhaustive, it is widely recognised and utilised [2, 18, 23–27]

Despite the availability of publicly-funded antenatal care services in India, the utilisation of these services among pregnant women remains dismally low. Notably, a nationwide cross-sectional survey conducted in 2015–16 found that only 21% of pregnant women received the recommended full antenatal care [28]. Despite significant improvements in maternal health outcomes in India in the last thirty years, certain states and subgroups of the population still seem to be off track in meeting the Sustainable Development Goal of reducing maternal mortality to below 70 by 2030 [29]. To tackle this pressing public health challenge, it is imperative to identify pregnant women at risk of missing full antenatal care and discern the underlying factors contributing to this issue. This critical information can then be leveraged to develop targeted and effective public health interventions to improve maternal health and reduce maternal mortality.

The literature indicates that the low utilisation of maternal healthcare services is a multifaceted issue influenced by various factors [18, 20, 23, 27]. These include limited access to healthcare facilities, particularly in rural areas, poverty, and low income, as well as inadequate awareness regarding maternal healthcare, including the significance of prenatal care and the potential risks associated with childbirth [30, 31]. Additionally, the lack of female empowerment and decision-making authority can adversely impact maternal healthcare utilisation [32]. Furthermore, cultural beliefs, attitudes, social stigma, and discrimination have been found to be influential factors [33]. Socioeconomic and educational status are also significant contributors to maternal healthcare utilisation. Studies show that women from higher socioeconomic backgrounds and those with higher education levels are more likely to access maternal healthcare services [19, 20, 27]. Moreover, limited transportation availability poses a challenge, particularly in geographically challenging areas, hindering access to care services. On the supply side, inadequate infrastructure and a shortage of trained staff can lead to suboptimal care and may discourage women from seeking care [30, 31, 34].

Adolescence is a critical period of physical, social, and emotional development, and pregnancy during this time can profoundly impact a young woman’s health and future. In addition, adolescent pregnancy is associated with an increased risk of maternal and fetal morbidity and mortality, making access to quality ANC services particularly important for this population [26]. However, adolescent girls are often subjected to discrimination and marginalisation in several societies, making accessing health services, including ANC, challenging [35]. This challenge can be further compounded by factors such as poverty, limited education, and lack of access to transportation, which can act as barriers to seeking care [35–37]. Furthermore, cultural norms and beliefs may discourage adolescent girls from seeking care, or they may feel embarrassed or ashamed to attend ANC visits [38–40].

Given the paramount importance of antenatal care (ANC) in mitigating the health risks associated with adolescent pregnancy, it is imperative to investigate the factors that affect the utilisation of full ANC services among adolescent women. This is particularly critical for SC/ST women, who have historically faced disadvantages in various aspects of life. However, despite the abundant literature on the utilisation of full ANC services in India, no studies have specifically examined the utilisation of these services among adolescent SC/ST women. Thus, this study aims to address this gap by investigating the utilisation of full ANC services among adolescent SC/ST women in India, using nationally representative data from the National Family Health Survey-4.

## Data and methods

### Data source

The data for this study came from the National Family Health Survey (NFHS-4), the fourth round of a large-scale, multi-round survey covering the whole country. It was conducted in 2015 and 2016. The NFHS is India’s version of the DHS and provides reliable estimates of national, state, and regional indicators, including maternal and child health care services. NFHS-4 was approved by the Ministry of Health and Family Welfare and conducted by the International Institute of Population Sciences (IIPS), Mumbai [41]. NFHS-4 employed a systematic multistage stratified sampling design. The survey’s sampling methodology is described in detail in the NFHS-4 national report.

**Fig. 1:**
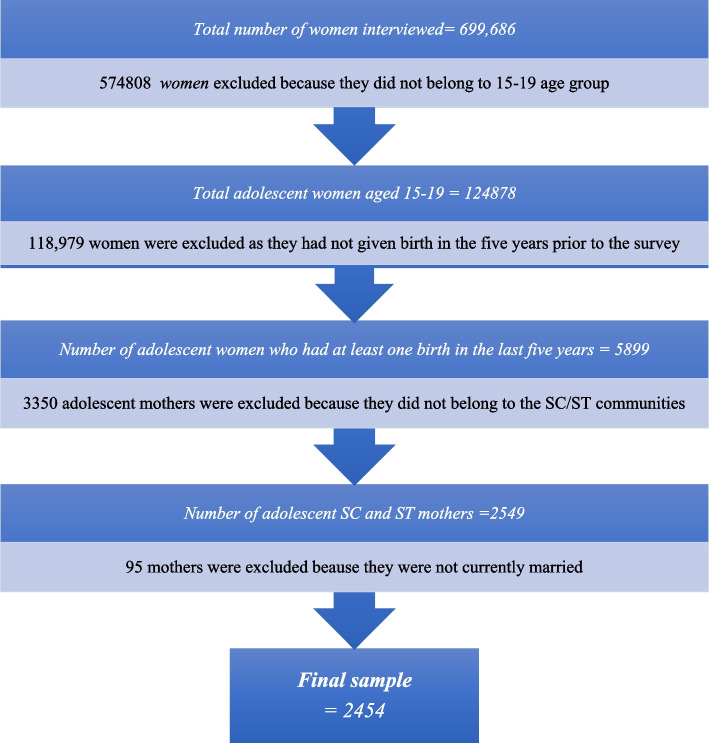
Flow chart showing the selection process of the study sample (currently married adolescent SC/ST mothers)

The survey conducted face-to-face interviews with 699,686 ever-married mothers aged 15–49 from 601,509 sampled households in India, yielding an overall response rate of 96.7%. Of these women, 17.84% (124,878) belonged to the 15–19 age group. After excluding 118,979 of these women (95.27%) who had not given birth in the five years preceding the survey, the study obtained a sample of 5,899 adolescent mothers, of which 43% (2,549) belonged to the SC and ST social groups. Furthermore, 3.7% (95) of the mothers were removed from the sample because they were not currently married. Therefore, the analysis in this paper is based on a sample of 2,454 SC/ST adolescent mothers (see Fig. 1).

### Dependent variable

In this study, the dependent variable used was “full ANC,” which comprised of three main components: having at least four ANC visits, receiving at least two tetanus toxoid (TT) injections, and consuming iron-folic acid (IFA) tablets or an equivalent amount of IFA syrup for 100 days during pregnancy. A pregnant mother was considered to have received “full ANC” only if she met all three criteria [42]. The “full ANC” indicator is recommended by the Ministry of Health and Family Welfare, the Government of India, and the World Health Organization [43], and is also used in the National Family Health Survey’s national and state level reports.

### Independent variables

We considered a range of socioeconomic and demographic predictors such as woman’s education, mothers’ occupation, religion, exposure to mass media, meeting with frontline worker, economic status, meeting with ASHA worker, parity, region of residence, and place of residence. The choice of these variables is guided by existing literature available from low- and middle-income developing countries on antenatal care utilisation [23, 44–53]. The model of the utilisation of maternal health services used in this analysis is based on the previous studies and models. (See Appendix A Table).

### Statistical analysis

We used both bivariate and multivariate analysis in order to identify factors associated with full ANC use among SC/ST adolescent mothers in India. Contingency table was used to understand how the utilisation of full ANC varied by socioeconomic and demographic characteristics of SC/ST adolescent mothers. Binary logistic regression was used to understand the net effect of predictor variables on the use of full ANC. We chose binary logistic regression because our response variable was dichotomous (i.e., binary) in nature. Before the final regression model was run with all independent variables included, we evaluated the relationship between the dependent variable and each individual independent variable through the use of a logistic regression. The odds ratios obtained from this analysis were referred to as “unadjusted” odds ratios since the models did not control for other variables. Those independent variables which did not turn out to be statistically significant were not included in the final regression model. Those variables that were found to have a statistically significant relationship were included in the final regression model and the odds ratios obtained from this analysis were referred to as “adjusted” odds ratios. These adjusted odds ratios were then utilised to conclude the effect of the independent variables on the dependent variable. The likelihood of multicollinearity impacting the results of our regression analysis was assessed using the variance inflation factor (VIF). The VIF is a measure of how much the variance of a regression coefficient is increased due to multicollinearity within the model. A general guideline suggests that VIF values above four warrant further examination, while VIF values exceeding ten indicate significant multicollinearity that requires correction. However, none of the VIF values for our regression model were above four indicating that multicollinearity was not a concern [54]. The results of the logistic regression models were presented in the form of odds ratios with *p*-values and 95% confidence intervals (CI). To accommodate the intricate survey design of NFHS-5, we incorporated the ‘svyset’ command in Stata16 [55].

## Results

Altogether, the sample for the entire country consisted of 5899 adolescent mothers, with approximately 43% (2549) belonging to the SC/ST social categories. This figure was slightly higher than the representation of SC/ST in the overall population, which is estimated to be around 25%. The increased fertility among SC/ST adolescent women as compared to non-SC/ST women may account for this discrepancy. Table 1 provides details regarding the distribution of the sampled SC/ST adolescent mothers based on their background characteristics.

**Table 1:** Percentage distribution of adolescent SC/ST mothers by background characteristics in India, NFHS 4 (2015–16)

Background characteristics	%	N
**Wealth index**		
Poorest	37.63	948
Poorer	32.84	799
Middle	19.36	441
Richer	8.09	201
Richest	2.08	65
**Religion**		
Hindu	91.05	1,940
Muslim	2.04	56
Others	6.91	458
**Mass media exposure**		
Not exposure	27.20	746
Low exposure	48.77	1,117
Medium exposure	18.49	454
High exposure	5.54	137
**Parity**		
1	85.36	2,086
2 or more	14.64	368
**Mother’s education**		
No education	23.63	606
Primary	15.20	387
Middle and above	61.17	1,461
**Mother’s current age**		
15–18 years	42.94	1,066
19 years	57.06	1388
**Met frontline health worker**		
No	41.18	1131
Yes	58.82	1323
**Residence**		
Urban	15.72	324
Rural	84.28	2,130
**Region**		
North	7.78	238
Central	16.92	545
East	41.42	721
North East	4.44	512
West	14.05	228
South	15.38	210
**Full ANC**		
No	82.2	2,097
Yes	17.8	357
**2 or more TT injections**		
No	17.11	501
Yes	82.89	1,953
**Taken IFA tablets/syrup for 100 or more days**		
No	72.5	1,845
Yes	27.5	609
**4 or more visit ANC**		
No	54.3	1,376
Yes	45.7	1,078

*N* number of mothers (frequency), All percentages are weighted

### Utilisation of full ANC and different components of full ANC

**Fig. 2:**
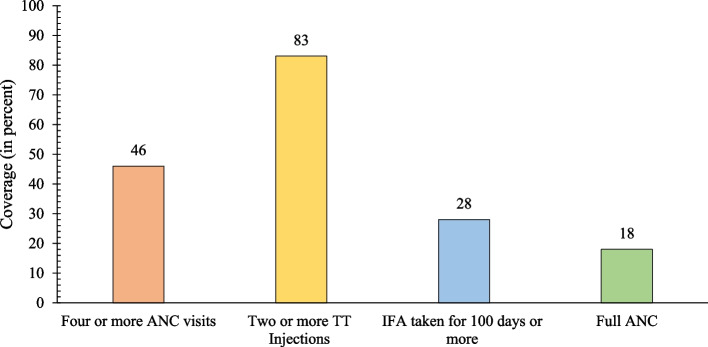
Utilisation of full ANC and different components of full ANC among adolescent SC/ST mothers in India, NFHS-4 (2015–2016)

Figure 2 shows that the utilisation of full ANC among Indian adolescent SC/ST mothers was inadequate, with only about 18% utilising the full ANC. Among the three components of full ANC, the coverage of two or more TT injections was relatively high, with 83% of adolescent SC/ST mothers reporting to have received them. In contrast, the utilisation of other two key components of full ANC was found to be low, with only 46% of adolescent SC/ST mothers having made four or more ANC visits, and only 28% consuming the recommended number of IFA tablets or equivalent amount of IFA syrup.

### Differentials in the utilisation of full antenatal care (ANC)

Table 2 displays the proportion of adolescent SC/ST mothers who reported receiving full ANC, categorised by their background characteristics. The results reveal significant discrepancies in full ANC utilisation across diverse categories of socioeconomic status, religion, region of residence, and maternal education. For instance, mothers in the poorest quintile were far less likely to receive full ANC, with only 10% reporting utilisation, compared to their counterparts in the richest quintile, where 40% reported utilisation. Furthermore, there was a notable gap in full ANC utilisation between Hindu and Muslim women, as only 17% of Hindu mothers received full ANC in comparison to a meagre 2% of Muslim mothers. The difference was most marked between adolescent SC/ST mothers residing in the central region (coverage of full ANC at 4%) versus those residing in the southern region (coverage of full ANC at 40%). Lastly, mothers who had met with a frontline worker reported almost twice the coverage of full ANC (21%) compared to those who had not (12%). Full ANC utilisation showed minor variation across other variables.

**Table 2:** Percentage of adolescent SC and ST mothers with full ANC by background characteristics in India, NFHS 4 (2015–16)

Background characteristics	Full ANC (in %)
**Wealth Index**	
Poorest	9.75
Poorer	20.23
Middle	24.90
Richer	22.49
Richest	40.89
**Religion**	
Hindu	17.35
Muslim	2.26
Others	28.30
**Mass media exposure**	
Not exposure	11.79
Low exposure	16.00
Medium exposure	29.43
High exposure	24.38
**Parity**	
1	18.53
2 or more	13.56
**Met frontline health worker**	
No	12.22
Yes	21.71
**Mother’s education**	
No education®	9.75
Primary	14.45
Middle and above	21.75
**Mother’s current age**	
15–18 years	16.12
19 years	19.07
**Place of residence**	
Urban	19.91
Rural	17.41
**Region**	
North	14.9
Central	4.17
East	15.02
Northeast	13.59
West	21.29
South	39.80

All percentages of full ANC are weighted

### Determinants of full antenatal care (ANC) utilisation

A multivariable logistic regression was used to examine the individual influence of various factors on SBI. The outcomes were presented as odds ratios. The unadjusted odds ratios showed that the mother’s age and parity were not significant predictors and were therefore not included in the final (adjusted) regression model (see Table 3). The final model revealed the wealth index, religion, place of residence, and interaction with a frontline worker were the statistically significant factors that determine the utilisation of full ANC services among adolescent SC and ST mothers, while mother’s education and mass media exposure turned insignificant (see Table 4).

**Table 3:** Unadjusted odds ratios and 95% confidence intervals for receiving full ANC among adolescent SC and ST mothers in India, NFHS (2015–16)

Background characteristics	Odds ratio	*p*-value	95% CI	
			**Lower**	**Upper**
**Wealth Index**				
Poorest®				
Poorer	2.347	< 0.001	1.543	3.568
Middle	3.068	< 0.001	1.928	4.881
Richer	2.685	<0.001	1.475	4.886
Richest	6.404	<0.001	2.182	18.791
**Religion**				
Hindu®				
Muslim	0.109	< 0.001	0.036	0.331
Others	1.879	0.048	1.006	3.509
**Mass media exposure**				
No exposure®				
Low exposure	1.425	0.097	0.938	2.164
Medium exposure	3.120	< 0.001	1.923	5.060
High exposure	2.413	0.012	1.217	4.779
**Parity**				
1®				
2 or more	0.689	0.123	0.430	1.105
**Met frontline health worker**				
No®				
Yes	1.992	< 0.001	1.374	2.887
**Mother’s education**				
No education®				
Primary	1.565	0.154	0.845	2.894
Middle and above	2.574	< 0.001	1.667	3.971
**Mother’s current age**				
15–18 year®				
19 years	1.226	0.230	0.878	1.708
**Place of residence**				
Urban®				
Rural	0.848	0.512	0.517	1.388
**Region**				
North®				
Central	0.248	< 0.001	0.132	0.466
East	1.009	0.974	0.591	1.721
Northeast	0.898	0.713	0.507	1.589
West	1.544	0.184	0.813	2.929
South	3.776	< 0.001	2.161	6.593

® - Reference category, *CI* - Confidence interval

**Table 4:** Adjusted odds ratios and 95% confidence intervals (CI) for receiving full ANC among adolescent SC and ST mothers in India, NFHS (2015–16)

Variables	Odds ratio	*p*-value	95% CI	
			**Lower**	**Upper**
**Wealth Index**				
Poorest®				
Poorer	1.776	0.016	1.111	2.838
Middle	1.933	0.026	1.084	3.446
Richer	1.674	0.133	0.854	3.279
Richest	5.505	0.003	1.804	16.800
**Religion**				
Hindu®				
Muslim	0.143	<0.001	0.044	0.459
Others	1.280	0.424	0.698	2.348
**Mass media exposure**				
No exposure®				
Low exposure	0.712	0.191	0.429	1.184
Medium exposure	1.285	0.412	0.706	2.339
High exposure	0.887	0.779	0.382	2.055
**Mother’s education**				
No education®				
Primary	1.274	0.472	0.659	2.465
Middle and above	1.377	0.230	0.816	2.324
**Met frontline health worker**				
No®				
Yes	1.821	0.002	1.241	2.670
**Region**				
North®				
Central	0.331	<0.001	0.168	0.652
East	1.375	0.312	0.742	2.549
Northeast	0.913	0.781	0.481	1.734
West	1.716	0.109	0.886	3.320
South	3.575	< 0.001	1.917	6.664

® - Reference category, *CI -* Confidence interval

Mothers from the richest wealth quintile were more than five times (OR = 5.505, CI = 1.804–16.800) more likely to receive full ANC than those belonging to the poorest wealth quintile. Mothers who had met with a frontline worker were nearly two times more likely (OR = 1.821, CI = 1.241–2.670) to receive full ANC than mothers who had not met with a frontline worker. Muslim mothers were far less likely (OR = 0.143, CI = 0.044–0.459) to receive full ANC than their Hindu counterparts. Adolescent SC and ST mothers residing in the South had almost four times higher odds of receiving full ANC (OR = 3.575, CI = 1.917–6.664) compared to their counterparts in the North. On the other hand, those living in the Central region had lower odds of receiving full ANC (OR = 0.331, CI = 0.168–0.652).

## Discussion

The purpose of this study was to examine the coverage of full ANC among SC/ST adolescent mothers and the factors associated with it. The study’s results indicate that the coverage of full utilisation of ANC services among adolescent mothers from these marginalised groups remains inadequate [56] The coverage of four or more ANC and sufficient intake of IFA tablets or syrup among the three components of full ANC was significantly lower than the coverage of TT injection. Several factors were found associated with the utilisation of full ANC services among SC/ST adolescent mothers. These included household wealth, religion, parity, region of residence, and interaction with frontline workers.

Among the three full ANC components, the uptake of IFA was considerably lower than other two components. Only 3 out of 10 mothers had adequate uptake of IFA supplementation. This situation exists even when the Government of India has been making efforts to improve IFA intake among mothers under the Reproductive, Maternal, Newborn, Child plus Adolescent Health (RMNCH + A) program [57], *Anaemia Mukt Bharat* [58], and the *National Iron* + *Initiative* [59]. Previous research from Rajasthan and Odisha has shown that adverse effects, unpleasant smell and taste, forgetfulness, and a lack of information about IFA from frontline health workers restrict the usage of IFA among pregnant women [60]. In addition to these demand-side obstacles, supply-side obstacles, such as stock-outs, hinder access to IFA supplements [61]. There is an immediate need to rethink the strategy to boost the use of IFA supplementation among adolescent SC/ST mothers.

Furthermore, the current study indicates that religion plays a significant role in determining the utilisation of full ANC services among adolescent SC/ST mothers, which is consistent with earlier research conducted in India [31, 62]. Specifically, the study found that Muslim women were less likely to utilise full ANC services in comparison to their Hindu counterparts. It is likely that religious beliefs and traditional practices specific to Muslim women may contribute to their lower utilisation of antenatal care services. Additionally, the lower levels of autonomy and awareness among adolescent Muslim and SC/ST women may be associated with their inadequate utilisation of full ANC services [12, 63, 64]. Adolescent SC/ST mothers belonging to the Muslim community may encounter various hurdles such as cultural stigma, discrimination, and socioeconomic constraints that impede their access to adequate maternal health care services. These findings underscore the significance of addressing religious and cultural barriers that obstruct Muslim women’s ability to utilise full ANC services. To overcome these challenges, there is a need for culturally sensitive and inclusive maternal health programs that cater to the specific needs of adolescent Muslim SC/ST women.

The study findings indicate a significant discrepancy in the utilisation of full antenatal care services among adolescent SC/ST mothers from distinct economic backgrounds. Mothers from wealthier households are more likely to receive full ANC as compared to those from poor households. This disparity between the rich and poor is consistent with earlier research studies conducted in India [25, 56, 65–68] and elsewhere [32, 69, 70]. Adolescent SC/ST mothers from poor households face numerous obstacles in accessing full antenatal care services. They are often less educated, unemployed, and socially isolated, which makes it challenging for them to avail of such care. Due to their limited resources, they also tend to overlook the significance of maternal health care services, including full ANC. As a result, they prioritise spending their limited resources on daily basic needs over maternal health care. This is particularly true for poor adolescent SC/ST women, who are often uneducated, unemployed, and detached from social networks, thus making them more challenging to reach with full ANC [71, 72].

This study those women who had met with a frontline worker were more likely to utilise full ANC services. This is in line with many previous studies [31, 51]. Auxiliary Nurse Midwife (ANM), Accredited Social Health Activist (ASHA), and *Anganwadi* workers are key in improving maternal healthcare utilisation [31]. These frontline providers offer health education, counselling, and services, especially in rural areas with limited access to healthcare. ANMs and ASHAs are trained professionals responsible for prenatal and postpartum care, regular check-ups, and essential maternal health services. *Anganwadi* workers, serving in rural communities, offer maternal and child health services, nutrition, and health education, raising awareness about maternal health issues and encouraging proper antenatal care [9, 26]. These frontline workers help improve maternal healthcare utilisation, reduce maternal and infant mortality rates, and ensure quality maternal healthcare for marginalised mothers [31, 51]. The low coverage of full ANC among SC/ST mothers calls for a rethinking of the strategies employed by ANM, ASHA, and *Anganwadi* workers to increase utilisation of full ANC.

This study also highlights disparities in the full ANC coverage among adolescent SC/ST mothers in different regions. Adolescent mothers from the South region are more likely to receive full ANC, which aligns with findings from previous studies conducted in India [23, 67, 73, 74]. The reason for this disparity may be due to various factors such as differences in maternal healthcare infrastructure, healthcare access, awareness of maternal health issues, cultural beliefs, and health-seeking behaviours among the mothers in the southern states compared to other regions of India. Further research is needed to determine the specific factors that contribute to this disparity and how it can be addressed to improve maternal healthcare access and utilisation of full antenatal care services across India.

This study has several limitations that must be taken into consideration when interpreting the results. Firstly, certain important variables were not incorporated into the analysis due to a high number of missing values. Secondly, the study only analysed the association between explanatory factors and full utilisation of ANC services and did not examine any causal relationships. Additionally, the decision-making process of both spouses regarding the use of healthcare services was not included due to the lack of available data in the study. The sample size of this study did not allow to examine the utilisation of full ANC among SC/ST women at state and district level which could be useful for policymakers. Lastly, it should be noted that conclusions about causality cannot be drawn from this study as the data used is cross-section in nature. The study relies solely on self-reported data from the National Family Health Survey (NFHS-4) and does not use additional objective sources to validate the information provided. While self-reported data raises some concerns, it is less likely to be biased in maternal healthcare-related events than in other sensitive topics like sexual behaviour.

## Conclusion

Despite various government initiatives, the utilisation of full ANC among adolescent SC/ST mothers remains inadequate and leaves much to be desired. This population of mothers has been largely neglected in maternal health policies and programs, despite being one of the most vulnerable groups of reproductive age mothers. The results of the study demonstrate that socioeconomic factors, including household wealth, religion, place of residence, and interaction with frontline workers, have an impact on the utilisation of full ANC among adolescent SC/ST mothers. These findings emphasise the importance of addressing social determinants of health to enhance full ANC utilisation among adolescent SC/ST mothers. Additionally, the critical role of frontline workers such as ANM, ASHA, and *Anganwadi* worker in improving full ANC coverage must be recognised. Furthermore, targeted interventions are needed to improve full ANC utilisation among specific subgroups of adolescent SC/ST mothers in India.

## Supplementary Information

**Additional file 1: Appendix Table A.** Operational definitions and categorization of variables used in the study.

## Data Availability

The dataset analysed during the current study is available in the Demographic and Health Surveys (DHS) repository, https://dhsprogram.com/data/available-datasets.cfm, and can be obtained for free by sending an online request. Codes used in the analysis can be made available from the corresponding author on request.

## Abbreviations

WHO: World Health Organization
NFHS: National Family Health Survey
OR: Odds Ratio
CI: Confidence Interval
SC: Scheduled Caste
ST: Scheduled Tribe
ANC: Antenatal Care
MMR: Maternal Mortality Ratio
RCH: Reproductive and Child Health
