# Supplementary material for: Assessing the coverage of full antenatal care among adolescent mothers from scheduled tribe and scheduled caste communities in India
Source: BMC Public Health. 2023 May 1;23:798. doi: 10.1186/s12889-023-15656-1 (PMC10150462; doi:10.1186/s12889-023-15656-1)
Supplement: Supplementary file 1 — Additional file 1: Appendix Table A. Operational definitions and categorization of variables used in the study. [file 12889_2023_15656_MOESM1_ESM.docx]

**Appendix Table A**

**Operational definitions and categorization of variables used in the study**

| Variables | Definitions |
| --- | --- |
| Mother's education | Mother's education is defined based on years of schooling and has four categories: Illiterate (0) zero years of schooling; Primary (1) 1–5 years of schooling; Secondary (2) 6–10 years of schooling; Above secondary (3) more than 10 years of schooling. |
| Religion | Religion has three categories – Hindu (1); Muslim (2); Others (3) all religious groups other than Hindu and Muslim. |
| Wealth Index | The wealth index is a composite measure that takes into account the household's assets and amenities, serving as an indicator of the household's socioeconomic status. It is commonly used as a surrogate for household income in studies utilizing data from NFHS and DHS. This index is typically divided into five categories: Poorest (0); Poorer (1); Middle (2); Richer (3); Richest (4). |
| Region of residence | A region in this study is a group of Indian states. North region (1) includes Jammu & Kashmir, Himachal Pradesh, Punjab, Rajasthan, Haryana, Chandigarh (Union Territory - UT), and Delhi; Central region (2) includes the states of Uttar Pradesh, Uttarakhand, Madhya Pradesh, and Chhattisgarh; East region (3) includes the states of Bihar, Jharkhand, West Bengal, and Odisha; North-East region (4) includes the states of Sikkim, Assam, Meghalaya, Manipur, Mizoram, Nagaland, Tripura, and Arunachal Pradesh; West region (5) includes the states of Gujarat, Maharashtra, Goa and UTs of Dadra & Nagar Haveli and Daman & Diu; South region (6) includes the states of Kerala, Karnataka, Andhra Pradesh, Tamil Nadu, Telangana and the UTs of Andaman & Nicobar Islands, Pondicherry) |
| Place of residence | Whether the respondent lives in a rural or urban area. This variable has two categories - Urban (0); Rural (1). |
| Parity | Parity is defined based on having several children by a mother. This variable is divided into two categories - 1 child (1); 2, 3, and 4 children (2) |
| Mass media exposure | Mass media exposure is defined based on the frequency of reading a newspaper/listening to the radio/watching television by mothers. The frequency of exposure is coded as; at least once a week or more (1); and less than once a week (0). |
| Meeting with a frontline health worker | This variable is related to whether the woman met with a frontline health worker (ASHA/*Angandwadi*/ANM) in the last three months. This variable has two categories – met a worker: Yes (1) and did not meet a worker: No (0) |
| Mother’s occupation | A mother’s occupation is defined based on her work. This variable is divided into two categories- Not working (0), and Working (1) |
| Full ANC | A woman is considered to have full ANC who has at least 4 ANC visits, 2 tetanus toxoid (TT) injections, and recommended quantity of iron-folic acid (IFA) tablets/syrup for 100 days or more during her pregnancy. It is a dichotomous variable with categories No (0) and Yes (1) |
| Tetanus toxoid (TT) | The number of TT injections taken during pregnancy – Zero and One (0); Two or more (1). |
| Iron and Folic Acid (IFA) tablets/syrup | Consumption of adequate IFA tablets/syrup for 100 days or more during pregnancy. It has two categories: No (0) and Yes (1). |
| Antenatal care (ANC) visits | The number of ANC visits by the mother during the entire duration of her pregnancy. It has two categories - No ANC visit and less than four visits (0); Four and more than four visits ANC (1) |
| Meeting with Anganwadi worker | This variable is related to meeting with an Anganwadi worker. This variable is divided into two categories – those who met with an Anganwadi worker: Yes (1) and those who had not met with an Anganwadi worker: No (0) |

**Note:** The state of Jammu and Kashmir was divided into two union territories in August 2019 because we are reporting finding for 2015-16, we report findings for the state of Jammu and Kashmir.
